# Lipocalin-2 Variants and Their Relationship With Cardio-Renal Risk Factors

**DOI:** 10.3389/fendo.2021.781763

**Published:** 2021-12-06

**Authors:** Dahui Li, Haoyun Li, Carlie Bauer, Yue Hu, Joshua R. Lewis, Aimin Xu, Itamar Levinger, Yu Wang

**Affiliations:** ^1^ The State Key Laboratory of Pharmaceutical Biotechnology, The University of Hong Kong, Hong Kong, Hong Kong SAR, China; ^2^ Department of Pharmacology and Pharmacy, The University of Hong Kong, Hong Kong, Hong Kong SAR, China; ^3^ Department of Medicine, The University of Hong Kong, Hong Kong, Hong Kong SAR, China; ^4^ Institute for Health and Sport, Victoria University, Melbourne, VIC, Australia; ^5^ Medical School, University of Western Australia, Perth, WA, Australia; ^6^ Institute for Nutrition Research, School of Medical and Health Sciences, Edith Cowan University, Joondalup, WA, Australia; ^7^ Centre for Kidney Research, Children’s Hospital at Westmead, School of Public Health, Sydney Medical School, The University of Sydney, Sydney, NSW, Australia; ^8^ Australian Institute for Musculoskeletal Science, Victoria University, University of Melbourne, Western Health, St. Albans, VIC, Australia

**Keywords:** Lipocalin-2 (Lcn2), protein variants, biomarker, heart, kidney

## Abstract

**Objectives:**

To investigate the serum, plasma and urine levels of lipocalin-2 (LCN2) variants in healthy humans and their associations with risk factors for cardiometabolic (CMD) and chronic kidney (CKD) diseases.

**Methods:**

Fifty-nine males and 41 females participated in the study. Blood and urine were collected following an overnight fasting. LCN2 variants were analyzed using validated in-house ELISA kits. Heart rate, blood pressure, lipids profile, glucose, adiponectin, high-sensitivity C-reactive protein (hsCRP), creatinine, cystatin C, and biomarkers for kidney function were assessed.

**Results:**

The levels of hLcn2, C87A and R81E in serum and urine, but not plasma, were significantly higher in men than women. Increased levels of LCN2 variants, as well as their relative ratios, in serum and plasma were positively associated with body mass index, blood pressure, triglyceride and hsCRP (*P*<0.05). No significant correlations were found between these measures and hLcn2, C87A or R81E in urine. However, LCN2 variants in urine, but not plasma or serum, were correlated with biomarkers of kidney function (*P*<0.05).

**Conclusions:**

Both the serum and plasma levels of LCN2 variants, as well as their ratios are associated with increased cardiometabolic risk, whereas those in urine are correlated with renal dysfunction. LCN2 variants represent promising biomarkers for CMD and CKD.

## Introduction

Emerging research reveals a complex and actively regulated crosstalk between bone, muscle and adipose tissue, which is implicated in metabolic and cardiovascular regulation (1). Lipocalin-2 (LCN2), also known as neutrophil gelatinase-associated lipocalin (NGAL), super inducible protein 24 (SIP24), 24p3/uterocalin, or α2-microglobulin-related protein/neu-related lipocalin, is a member of the lipocalin protein family characterized by highly diversified patterns of expression and structure-function relationships (2). LCN2 is expressed and released from various cell types (3), and may represent a specific hormone involved in the interactions between bone, muscle and adipose tissue (1). Adipose-derived LCN2 is highly upregulated in humans and animal models of obesity (4), contributing to the regulation of local, regional and systemic inflammation, immunity, and metabolism (5). The pathological forms of adipose-derived LCN2 play a causal role in obesity-associated cardiometabolic and renal diseases (6, 7).

Although the serum, plasma and urine levels of LCN2 have been regarded as biomarkers for cardiometabolic (CMD) and chronic kidney (CKD) diseases (4, 8–11), there are inconsistent reports on the use of this molecule as a biomarker for early diagnosis or prognosis (8, 12, 13). Due to the dynamic ligand-binding, posttranslational modification and protein-protein interaction, LCN2 exists as multiple variants that are differentially expressed in tissues and organs and present in blood circulation (14, 15). Previous studies have identified three LCN2 variants, including the polyaminated hLcn2, non-polyaminated C87A, and R81E with altered ligand binding activity (14–16). Different LCN2 variants are distinctively present in samples from healthy humans and patients with cardiometabolic abnormalities (4, 14, 15), and play diversified roles in causing cardiovascular, renal and metabolic abnormalities (14–18). However, the vast majority of previous research focus on total LCN2 levels.

To date no studies have investigated the associations between LCN2 variants in serum, plasma and urine of the same individuals and their correlations with pathophysiological processes or diseases. It is not clear whether there are differences in LCN2 variants between males and females and whether they correlate to different indices of cardiometabolic and renal function. The aims of the present study were to investigate LCN2 variants in serum, plasma and urine in apparently healthy individuals and identify the associations between their levels and risk factors for CMD and CKD.

## Materials and Methods

### Participants

All experiments were performed in accordance with institutional guidelines and the principles outlined in the Declaration of Helsinki. Written informed consent was obtained from all participants prior to data (medical records) and sample collection. One hundred volunteers (59 males and 41 females) were recruited from the community in Hong Kong between October 2015 and June 2016 for studies approved by the Institutional Review Board of the University of Hong Kong/Hospital Authority Hong Kong West Cluster (reference number UW 14-044) (14, 15). Exclusion criteria included pregnancy or lactation; alcohol intake within the past 24 hour; long-term drug treatment or medications taken within one week prior to the study; and diagnosis of hypertension, diabetes, dyslipidaemia, anaemia, coronary heart disease, chronic obstructive pulmonary disease, asthma, hepatitis, primary hyperaldosteronism, renal dysfunction and eczema. Age, sex, weight, height, heart rate, systolic (SBP) and diastolic (DBP) blood pressure were recorded by standard procedures. Body mass index (BMI) was calculated using recorded weight and height. Serum, plasma and urine samples from healthy volunteers were collected between 8:00 a.m. and 10:00 a.m. after overnight fasting for 10-12 hours (14). All samples were stored at -80°C until analyses. The clinical characteristics as well as the biomarker levels of the participants are summarized in **Table 1**. The estimated glomerular filtration rate (eGFR) was calculated using the CKD-Epidemiology Collaboration formula (19).

**Table 1:** Characteristics of the study participants.

	Whole cohort (n = 100)	Males (n = 59)	Females (n = 41)
Age (years)	48.2 ± 7.3	48.2 ± 7.4	48.3 ± 7.1
BMI (kg/m^2^)	23.8 ± 2.7	24.7 ± 2.1	22.7 ± 3.0
Heart rate	65.4 ± 8.1	65.5 ± 8.5	65.1 ± 7.7
SBP (mmHg)	117.0 ± 13.9	120.8 ± 12.8	111.4 ± 13.5
DBP (mmHg)	74.5 ± 10.6	77.9 ± 9.7	69.6 ± 10.0
TG (mmol/L)	1.7 ± 0.8	1.8 ± 0.7	1.6 ± 0.7
TC (mmol/L)	5.4 ± 0.9	5.4 ± 0.9	5.5 ± 0.9
HDLc (mmol/L)	1.6 ± 0.2	1.6 ± 0.2	1.6 ± 0.2
LDLc (mmol/L)	3.5 ± 0.9	3.4 ± 0.8	3.6 ± 0.9
FBG (mmol/L)	5.8 ± 0.7	5.8 ± 0.7	5.7 ± 0.6
Adiponectin(mg/L)	17.9 ± 11.2	13.1 ± 7.5	24.8 ± 12.2
hsCRP(mg/L)	6.2 ± 9.6	6.9 ± 10.8	5.1 ± 7.5
sAldo (nmol/L)	72.4 ± 223.7	96.0 ± 291.1	39.7 ± 32.8
sCr (µmol/L)	52.5 ± 9.6	55.3 ± 9.5	48.4 ± 8.3
sCysC (mol/L)	19.4 ± 9.6	22.6 ± 9.6	14.9 ± 7.7
uAlb (g/L)	3058.8 ± 2063.6	2875.8 ± 2077.1	3322.1 ± 2040.4
uAldo (nmol/L)	539.1 ± 790.4	615.4 ± 868.3	423.9 ± 649.1
uHap (mmol/L)	0.4 ± 0.8	0.4 ± 1.0	0.3 ± 0.5
uKIM-1 (µmol/L)	13.4 ± 24.5	17.8 ± 30.1	7.2 ± 9.9
uCr (µmol/L)	7714.5 ± 6885.4	9410.7 ± 7600.6	5273.6 ± 4818.7
ACR (mg/mmol)	0.2 ± 0.4	0.1 ± 0.3	0.3 ± 0.6
eGFR^a^ (mL/min/1.73m^2^)	115.1 ± 10.5	117.6 ± 10.5	111.6 ± 9.5

a Data are shown as means ± SD.

ACR, albumin-to-creatinine ratio; BMI, body mass index; DBP, diastolic blood pressure; eGFR, estimated glomerular filtration rate; FBG, fasting blood glucose; HDLc, high-density lipoprotein cholesterol; hsCRP, high-sensitivity C-reactive protein; LDLc, low-density lipoprotein cholesterol; TC, total cholesterol; TG, triglycerides; sAldo, serum aldosterone; sCr, serum creatinine; SBP, systolic blood pressure; uAlb, urinary albumin; uAldo, urinary aldosterone; uCr, urinary creatinine; uHap, urinary haptoglobin; uKIM-1, urinary kidney injury molecule-1.

### Laboratory Analyses

The blood glucose was analyzed using Accu-Chek Advantage II Glucometer (Roche Diagnostics, Mannheim, Germany). The circulating lipids including triglyceride, total cholesterol (TC), high (HDLc)- and low (LDLc)-density lipoprotein cholesterol were measured using LiquiColor test kits from Stanbio Laboratory (Boerne, TX, USA).

### Immunoassays

Plasma and serum concentrations of adiponectin, high-sensitivity C-reactive protein (hsCRP) was measured using in-house ELISA kits (https://www.antibody.hku.hk/ELISA.php) as described previously (14). Aldosterone levels in serum (sAldo) and urine (uAldo) samples were measured using the DetectX Aldosterone Enzyme Immunoassay kit (Cayman Chemical, Ann Arbor, MI, USA). The amount of creatinine was assayed in serum (sCr) and urine (uCr) samples using ELISA kit (catalog # ab65340) from Abcam (Cambridge, UK). Serum cystatin C (sCysC) was measured using Olympus AU400 from Olympus America Inc (NY, USA) as per manufacturer’s protocol. Albumin was determined in urine samples using ELISA kit (catalog # 1004) from Exocell Inc (Philadelphia, PA, USA). Haptoglobin in urine was examined by ELISA kit (catalog # RD191407100R) purchased from Biovendor (Candler, NC, USA). The urine levels of kidney injury molecule-1 (KIM-1) were measured using ELISA kit (catalog # DKM-100) purchased from R&D Systems (Minneapolis, MN, USA).

### Quantification of LCN2 Variants

The recombinant human LCN2 variants, including hLcn2, C87A and R81E, were expressed in and purified from *E. coli* as described (14–16). In brief, the prokaryotic plasmids including pPRO-HishLCN2, pPRO-His-hLCN2-C87A and pPRO-His-hLCN2-R81E were constructed and transformed into BL21 Competent cells (catalogue # 200131; Agilent Technologies, Santa Clara, CA, USA) to express hLcn2, C87A and R81E, respectively. All the recombinant proteins contained an NH_2_-terminal polyhistidine tag for affinity purification using the Ni-NTA Agarose from QIAGEN (Hilden, Germany). Endotoxin removal was performed using the Detoxi Endotoxin-Removal Gel (catalogue # 88272; Pierce Biotechnology Inc., Rockford, IL, USA) and confirmed by the Chromogenic Endotoxin Quant Kit (catalogue # 88282; Pierce Biotechnology Inc.). The purity of individual LCN2 variants was examined by SDS-PAGE, Western blotting and Ultra Performance Liquid Chromatography (UPLC; *ACQUITY UPLC^®^ System*, Waters, MA, USA) using a Bio SEC-3 HPLC column (catalogue # 5190-2503; 3µm, 150 Å, 4.6x300mm; Agilent, CA, USA).

The polyclonal antibodies against hLcn2, C87A or R81E were produced in New Zealand female rabbits, purified by affinity chromatography and applied to establish the respective sandwich ELISA kits for measuring the LCN2 variants (4, 14, 15). In brief, serum and plasma were both diluted at a ratio of 1:80, whereas urine samples were diluted at a ratio of 1:20. One hundred microliter samples or recombinant protein standards were added to each well of the coated ELISA plates and incubated for one hour at room temperature. After washing for three times and another one hour of incubation with the biotinylated antibodies, the streptavidin-conjugated horseradish peroxidase and substrates were added to detect the immune complexes. The reactions were stopped before measurement of the absorbance at 450 nm with a plate reader (BioTek Instruments Inc., Winooski, VT, USA).

### Statistical Analysis

All analyses were performed using the IBM SPSS Statistical Package version 27.0 software (SPSS Inc., Chicago, IL, USA). Data are expressed as means ± SD, medians with interquartile range or number as appropriate. The Kolmogorov–Smirnov test was used to analyze the distribution of different variables. ﻿Spearman’s rank correlation was performed to establish the degree of association between variables. The difference in concentrations of LCN2 variants between groups was evaluated using Mann-Whitney U test. Non-parametric Kruskal-Wallis H test was used for multiple comparisons. Multivariate logistic regression was computed to evaluate the combined influence of biomarkers on LCN2 variants.

## Results

**Figure 1:**
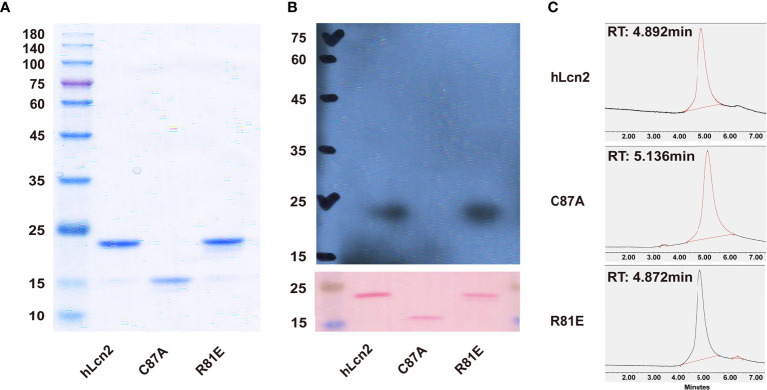
Comparison of the biochemical features of LCN2 variants. **(A)** After endotoxin removal, the recombinant proteins were separated by SDS-PAGE and stained with Coomassie Brilliant Blue. **(B)** The contents of polyamines in each of the LCN2 variants were examined by Western blotting using the antibody against spermidine. The membrane was stained with Ponceau S to confirm the protein loading (0.5 μg/lane). **(C)** UPLC was performed to analyze the three LCN2 variants. In brief, 10 μl of hLcn2, C87A and R81E protein solution (1µg/µl) was injected for size-exclusion chromatography at a flow rate of 0.5 ml/min. The eluted protein peaks were detected at 254 nm by a UV detector. The retention time (RT) was recorded for comparison.

In mammals, lipocalin-2 protein exists as variants that possess diversified structures and functions (3). The LCN2 variants, including hLcn2, C87A and R81E, were expressed and purified from *E. coli* (**Figure 1**). C87A migrated as a lower molecular weight species in SDS-PAGE and was eluted by size-exclusion chromatography at a retention time much later than those of R81E and hLcn2 (**Figure 1**). The R81E variant contained a higher amount of polyamine and was eluted earlier than those of hLcn2 and C87A (**Figure 1**).

Specific sandwich ELISA kits were developed in-house for measuring the three LCN2 variants in human samples (4, 14, 15). Fifty-nine males (age 48.2 ± 7.4 years and BMI 24.7 ± 2.1 kg/m^2^) and 41 females (age 48.3 ± 7.1 years and BMI 22.7 ± 3.0 kg/m^2^) were recruited for the study. Participant characteristics are displayed in **Table 1**. Concentrations of LCN2 variants and the differences between males and females are shown in **Table 2**. The levels of hLcn2, C87A and R81E in both serum and urine were significantly higher in men than women, same as the ratios of all variants (*P*<0.05). The ratios of urinary LCN2/uCr exhibited similar gender differences as those of urine LCN2 (**Table 2**). No significant difference between sexes were observed for LCN2 variants in plasma (*P*>0.05).

**Table 2:** Concentrations of LCN2 variants in samples from healthy cohort^a^.

	Plasma			Serum			Urine		
	Whole cohort	Male	Female	Whole cohort	Male	Female	Whole cohort	Male	Female
hLcn2 (ng/ml)	49.1(40.1-58.4)	50.0(41.3-58.3)	46.6(37.7-58.6)	71.1(43.4-139.7)	87.7(48.6-152.7)	58.1(34.2-100.2)^*^	68.4(30.0-177.7)	161.0(35.4-341.4)	33.9(17.3-105.6)^*^
C87A (ng/ml)	47.6(38.1-62.9)	47.3(40.5-62.9)	47.7(35.7-61.7)	93.8(63.1-143.2)	102.6(69.6-162.0)	84.2(54.6-113.1)^*^	**3.9(1.8-9.1)^#^**	**7.6(3.4-15.2)^#^**	3.0(1.6-5.0)^*#^
R81E (ng/ml)	39.0(31.8-48.1)	39.2(33.8-46.5)	35.3(30.5-49.9)	**99.4(82.1-115.9)^#^**	101.1(89.1-117.7)	92.4(78.1-112.8)^*#^	**4.4(2.8-10.2)^#^**	**7.3(4.2-14.5)^#^**	3.6(2.5-7.6)^*#^
	**Plasma/Urine**			**Serum/Urine**			Urinary LCN2/uCr*10^2^		
	Whole cohort	Male	Female	Whole cohort	Male	Female	Whole cohort	Male	Female
hLcn2	0.9(0.2-2.4)	1.4(0.4-2.9)	0.4(0.2-1.5)^*^	1.2(0.4-4.2)	1.6(0.8-6.7)	0.5(0.2-1.9)^*^	9.4(3.5-32.9)	5.1(2.3-15.0)	31.6(9.4-152.3)^*#^
C87A	**11.4(4.6-25.3)^#^**	**14.2(8.5-34.8)^#^**	5.8(3.2-19.8)^*#^	**23.7(9.6-52.7)^#^**	**28.1(20.6-86.9)^#^**	11.8(5.8-30.4)^*#^	**0.7(0.3-1.9)^#^**	**0.4(0.2-0.8)^#^**	1.7(0.7-6.3)^*#^
R81E	**7.1(3.6-14.1)^#^**	**10.4(4.-16.3)^#^**	5.0(2.3-9.5)^*#^	**20.6(9.7-35.5)^#^**	**25.2(12.3-38.9)^#^**	12.9(7.1-22.3)^*#^	**0.7(0.3-1.9)^#^**	**0.5(0.2-1.0)^#^**	1.7(0.8-6.9)^*#^

a Data are shown as median (interquartile range) values.

^*^P < 0.05 vs male subjects (Mann-Whitney U test); ^#^P < 0.05 vs hLcn2 (Kruskal-Wallis H test). Bold values, show significance after statistical analysis.

For the whole cohort, all three LCN2 variants correlated between plasma and serum (**Table 3**). The correlations were also present in each sex separately. In men, there were no correlations between plasma and urine or serum and urine levels of the three variants. In females, hLcn2 and C87A in the plasma were positively associated with those in the urine. There was also a correlation between serum and urine C87A in females (**Table 3**).

**Table 3:** Correlations between LCN2 variants in different samples.

LCN2 variant	plasma vs serum	plasma vs urine	serum vs urine
	**Total**		
hLcn2	0.412^c^	0.024	-0.036
C87A	0.496^c^	0.078	-0.040
R81E	0.375^c^	-0.174	-0.053
	**Male**		
hLcn2	0.330^a^	-0.233	-0.137
C87A	0.455^c^	-0.239	-0.172
R81E	0.231	-0.262	-0.124
	**Female**		
hLcn2	0.475^b^	0.424^b^	0.238
C87A	0.563^c^	0.503^b^	0.317^a^
R81E	0.586^c^	-0.003	-0.048

Spearman’s correlation was performed for analyses.

a P < 0.05.

b P < 0.01.

c P < 0.001.

Bold values, show significance after statistical analysis.

**Table 4** included the correlations of individual LCN2 variants in plasma, serum and urine with cardiometabolic risk factors and renal function. **Table 5** shows the correlations of the ratios of LCN2 variants with these parameters. Overall, higher levels of plasma or serum LCN2 variants were associated with a higher BMI, blood pressure, heart rate, TG and hsCRP (all *P*<0.05, **Table 4**). No significant correlations were found between urine hLcn2, C87A or R81E and these measures. However, urine hLcn2, C87A or R81E, but not their levels in plasma or serum, were correlated with biomarkers of kidney function including uAldo, uAlb, uHap, uKIM-1 and uCr (**Table 4**). A higher hLcn2/R81E and C87A/R81E ratio in the serum was correlated with higher BMI, DBP, HR, TG, hsCRP and lower adiponectin levels (**Table 5**). The ratio of hLcn2/C87A in the urine was correlated with measures of renal function, but not other risk factors (**Table 5**). After normalization to the creatinine levels, the urinary LCN2/uCr ratios were correlated with BMI, SBP, DBP, as well as measures of renal function (**Table 6**).

**Table 4:** Correlations between LCN2 variants and study variables.

	Plasma hLcn2	Plasma C87A	Plasma R81E	Serum hLcn2	Serum C87A	Serum R81E	Urine hLcn2	Urine C87A	Urine R81E
**BMI**	0.211^a^	0.159	0.186	0.419^c^	0.438^c^	0.292^b^	-0.060	-0.086	-0.099
**SBP**	0.191	0.204^a^	0.248^a^	0.164	0.191	0.152	-0.082	-0.108	-0.003
**DBP**	0.321^b^	0.322^b^	0.317^b^	0.289^b^	0.336^b^	0.253^a^	-0.122	-0.132	-0.070
**Heart rate**	0.291^b^	0.276^b^	0.202^a^	0.288^b^	0.295^b^	0.094	0.031	0.049	0.058
**TG**	0.284^b^	0.264^b^	0.379^c^	0.296^b^	0.366^c^	0.249^a^	0.006	0.009	0.020
**hsCRP**	0.282^b^	0.253^a^	0.297^b^	0.470^c^	0.461^c^	0.324^b^	0.082	0.077	0.007
**Adiponectin**	-0.221^a^	-0.181	-0.133	-0.386^c^	-0.351^c^	-0.320^b^	0.100	0.139	0.038
**uAldo**	0.146	0.208^a^	0.068	0.063	0.115	-0.061	0.335^b^	0.282^b^	0.352^b^
**uAlb**	-0.088	-0.151	-0.054	-0.024	-0.042	-0.057	-0.444^c^	-0.399^c^	-0.288^b^
**uHap**	-0.058	-0.050	-0.167	-0.084	-0.081	-0.03	0.201^a^	0.205^a^	0.236^a^
**uKIM-1**	0.061	0.093	0.034	0.077	0.125	0.047	0.254^a^	0.199	0.231^a^
**uCr**	0.094	0.127	0.007	0.137	0.175	0.050	0.223^a^	0.165	0.222^a^
**ACR**	0.002	-0.054	0.110	-0.054	-0.031	0.001	-0.242^a^	-0.201	-0.194
**eGFR**	-0.004	-0.020	0.007	-0.061	-0.062	-0.002	-0.009	-0.003	-0.024

Spearman’s correlation was performed for analyses.

a P < 0.05.

b P < 0.01.

c P < 0.001.

Bold values, show significance after statistical analysis.

**Table 5:** Correlations between the LCN2 variants ratios and cardiometabolic risk factors and renal function.

	Plasma/Urine			Serum/Urine			Plasma			Serum			Urine		
	hLcn2	C87A	R81E	hLcn2	C87A	R81E	hLcn2/C87A	hLcn2/R81E	C87A/R81E	hLcn2/C87A	hLcn2/R81E	C87A/R81E	hLcn2/C87A	hLcn2/R81E	C87A/R81E
BMI	0.087	0.118	0.122	0.243^a^	0.253^a^	0.119	0.03	-0.024	-0.06	0.317^b^	0.397^c^	0.390^c^	-0.002	-0.065	-0.081
SBP	0.113	0.149	0.066	0.17	0.171	0.013	-0.117	-0.117	0.027	0.092	0.13	0.139	-0.042	-0.158	-0.176
DBP	0.179	0.229^a^	0.148	0.253^a^	0.256^a^	0.087	-0.143	-0.08	0.085	0.143	0.237^a^	0.279^b^	-0.072	-0.136	-0.08
Heart rate	0.003	0.005	0.015	0.101	0.067	-0.043	-0.086	0.047	0.081	0.238^a^	0.302^b^	0.331^b^	-0.03	0.001	0.006
TG	0.031	0.04	0.043	0.141	0.138	0.033	-0.032	-0.189	-0.139	0.135	0.253^a^	0.319^b^	-0.058	-0.059	0.011
hsCRP	-0.042	-0.009	0.067	0.142	0.114	0.063	-0.073	-0.102	-0.027	0.398^c^	0.456^c^	0.410^c^	0.076	0.14	0.177
Adiponectin	-0.13	-0.185	-0.038	**-0.281^b^**	**-0.278^b^**	-0.08	-0.001	-0.103	-0.109	-0.349^c^	-0.363^c^	-0.300^b^	-0.012	0.024	0.095
uAldo	-0.127	-0.096	-0.094	-0.266^a^	-0.215^a^	-0.1	-0.054	0.014	0.036	-0.252^a^	-0.278^b^	-0.226^a^	0.186	0.14	0.112
uAlb	0.421^c^	0.341^b^	0.288^b^	0.348^c^	0.311^b^	0.271^a^	0.116	-0.013	-0.167	0.035	-0.016	-0.022	-0.395^a^	0.072	0.276^a^
uHap	-0.211^a^	-0.243^a^	-0.260^a^	-0.191	-0.222^a^	-0.226^a^	0.053	0.219^a^	0.169	-0.05	-0.064	-0.073	0.085	-0.052	-0.148
uKIM-1	-0.215^a^	-0.144	-0.224	-0.154	-0.1	-0.232^a^	-0.072	-0.064	0.068	0.003	0.083	0.12	0.326^b^	-0.072	-0.329^b^
uCr	-0.198^a^	-0.096	-0.225^a^	-0.11	-0.045	-0.224^a^	-0.09	0.06	0.185	0.055	0.169	0.229^a^	0.334^b^	-0.094	-0.383^c^
ACR	0.277^b^	0.185	0.234^a^	0.193	0.141	0.224^a^	0.092	-0.07	-0.196	-0.021	-0.119	-0.164	-0.349^c^	0.11	0.355^b^

Spearman’s correlation was performed for analyses.

a P < 0.05.

b P < 0.01.

c P < 0.001.

Bold values, show significance after statistical analysis.

**Table 6:** Correlations between the urinary LCN2/uCr ratios and cardiometabolic risk factors and renal function..

	urinary LCN2/uCr		
	hLcn2	C87A	R81E
BMI	-0.275^b^	-0.294^b^	-0.288^b^
SBP	**-0.208^a^**	-0.195	-0.141
DBP	-0.284^b^	-0.268^b^	-0.231^a^
Heart rate	0.024	0.070	0.016
TG	-0.086	-0.062	-0.042
hsCRP	-0.022	-0.043	-0.106
Adiponectin	0.326^b^	0.364^c^	0.301^b^
uAldo	-0.309^b^	-0.506^c^	-0.373^c^
uAlb	0.070	0.226^a^	0.211
uHap	0.099	0.078	0.051
uKIM-1	-0.273^a^	-0.408^c^	-0.341^b^
uCr	-0.477^c^	-0.663^c^	-0.554^c^
ACR	0.363^c^	0.542^c^	0.476^c^
eGFR	-0.055	-0.091	-0.070

Spearman’s correlation was performed for analyses.

a P < 0.05.

b P < 0.01.

c P < 0.001.

Bold values, show significance after statistical analysis.

## Discussion

LCN2 is a secreted glycoprotein implicated in a wide range of pathophysiological processes and energy metabolism (3). Elevated LCN2 levels contribute to the development of obesity-related medical complications and other pathologies, including CMD and CKD (7, 20). LCN2 is post-translationally modified by polyamination, which enhances the clearance of this protein from the circulation (16). By contrast, non-polyaminated LCN2 [represented by C87A in the present study] accumulates in tissues to cause injury and is implicated in the pathogenesis of obesity-associated cardiovascular complications (14, 16, 20). LCN2 interacts with the ferric-catecholate complex *via* the positively charged side chains of three amino acid residues, R81, K125 and K134 (21, 22). In the present study, R81E represents a variant of LCN2 with reduced binding affinity for the hydrophilic ligand, including the catecholate-type siderophores (23, 24). The weakened interactions with R81E may lead to the degradation of siderophore ligands or its selective association with catechols, in turn affecting the subsequent binding with LCN2 receptors such as megalin located at the apical membrane of the proximal tubules (25, 26).

The present study measured the concentrations of the three different LCN2 variants in bio-fluid samples from healthy individuals, assessed their associations with various cardiometabolic risk factors and kidney function. We reported that a) men have higher levels of hLcn2, C87A and R81E in serum and urine, but not plasma, than women, b) increased serum and plasma concentrations of hLcn2, C87A and R81E are positively associated with risk factors of CMD, but not CKD, while their levels in urine are related to biomarkers of kidney function. In women, but not in men, there were significant correlations between circulating and urine levels of LCN2 variants.

The results of the present study demonstrate substantial differences in concentrations of the LCN2 variants between serum, plasma and urine. Moreover, there appears a clear sex difference in urine and serum but not plasma levels of LCN2 variants. It was previously found that plasma LCN2 was higher in males compared to females (27, 28). This may be explained by sample size or ethnographic differences. While past studies report lower LCN2 in females, consistent with the present study, there is evidence that age further influences LCN2 levels in males and females (28). Further research is required to quantify the impact of age on the sex differences in LCN2 variants. In addition, LCN2 is known to be present in blood cells, including those of the erythroid, granuloid and monocyte/macrophage lineages (29, 30), and associated with blood coagulation (31–33), a process known to be significantly affected by gender. In mice, a sex-specific function of adipose-secreted LCN2 is implicated in metabolic regulation (34), suggesting that the ramifications of sex differences in LCN2 variants extend beyond their variances in concentration.

High levels of LCN2 are independently associated with a worse risk profile and all-cause mortality of coronary heart disease (35–37). The present results demonstrated that there were significant correlations between LCN2 variants in the serum or plasma and cardiometabolic risk factors. Moreover, the ratios of hLcn2/R81E and C87A/R81E in the serum were correlated with cardiometabolic risk factors, suggesting that LCN2 variants represent important biomarkers for risk stratification. As the circulating levels of LCN2 are affected by the changes in eGFR (38, 39), simultaneously monitoring different LCN2 variants and calculating their relative compositions could be a better and an effective approach for risk assessment in healthy populations. Future research investigating associations between LCN2 variants and cardiometabolic risk factors in human patients will be useful to establish the rationale for the use of LCN2 variants as biomarkers for CMD.

The present study showed that urine LCN2 variants were not significantly correlated with cardiometabolic variables, including BMI, heart rate, blood pressure, TG, hsCRP and adiponectin. LCN2 has been identified as a potential biomarker for kidney function outcomes (10). LCN2 is a small 25 kDa protein filtered by glomeruli and reabsorbed by the proximal tubules in kidney (40). In the present study, none of the LCN2 variants in plasma, serum or urine were significantly correlated with eGFR. However, significant correlations were found between urinary LCN2 variants and other markers of kidney function. The uAldo, uAlb and uHap were associated with all LCN2 variants in urine; the uKIM-1 and uCr were associated with hLcn2 and R81E in urine; and the ACR was associated with hLcn2 in urine. Considering the influences from variable urine volume, the urinary LCN2 variants were normalized to creatinine and the ratios significantly correlated with BMI, DBP, adiponectin, uAldo, uKIM-1 and ACR.

The study has limitations: First, the sample size was relatively small, and a large epidemiological study should be performed to validate the present results. Second, the study included only healthy individuals and the LCN2 variants in the serum, plasma and urine should be explored in human subjects with metabolic, cardiovascular, and renal diseases. Third, longitudinal investigations are warranted to investigate the diagnostic and prognostic values of LCN2 variants in different types of biofluids. Lastly, since the ethnicity may affect the risk for CMD and CKD, the research findings may not be generalized to other ethnic populations. The work may be used as a reference for future research but the results should be interpreted according to the enrolled subjects and limitation of collected data.

In conclusion, LCN2 variants in the serum and plasma as well their ratios are associated with increased risk factors of CMD, while their levels in the urine are correlated with biomarkers of kidney function. Monitoring LCN2 variants in the serum/plasma and urine may be used to identify people who are at risk for cardiometabolic renal diseases. LCN2 variants may represent potential specific therapeutic targets that warrant investigations.

## Data Availability Statement

The original contributions presented in the study are included in the article/supplementary material. Further inquiries can be directed to the corresponding author.

## Ethics Statement

The study was conducted according to the guidelines of the Declaration of Helsinki, and ap-proved by the Institutional Review Board of the University of Hong Kong/Hospital Authority Hong Kong West Cluster (reference number UW 14-044). The patients/participants provided their written informed consent to participate in this study.

## Author Contributions

DL and YH performed the measurements. YW and AX were involved in planning and supervised the work. DL and HL processed the experimental data. JL and CB performed the analysis. DL and YW drafted the manuscript and designed the figures. YW, IL, and JL aided in interpreting the results and worked on the manuscript. All authors discussed the results and commented on the manuscript, agreed to the published version of the manuscript.

## Funding

This work was supported by the grants from Seeding Funds for Basic Research of the University of Hong Kong, the General Research Funds (17117017 and 17121714) and Collaborative Research Funds (C7037-17W) of Research Grant Council, the Areas of Excellence Scheme (AoE/M-707/18) of University Grants Committee, and the NSFC-NHMRC Joint Research Scheme (113592/APP1113592). The salary of JL is supported by a National Heart Foundation of Australia Future Leader Fellowship (ID: 102817).

## Conflict of Interest

The authors declare that the research was conducted in the absence of any commercial or financial relationships that could be construed as a potential conflict of interest.

## Publisher’s Note

All claims expressed in this article are solely those of the authors and do not necessarily represent those of their affiliated organizations, or those of the publisher, the editors and the reviewers. Any product that may be evaluated in this article, or claim that may be made by its manufacturer, is not guaranteed or endorsed by the publisher.

## Abbreviations

ACR, albumin-to-creatinine ratio; BMI, body mass index; CKD, chronic kidney disease; CMD, cardiometabolic disease; CVD, cardiovascular disease; DBP, diastolic blood pressure; eGFR, estimated glomerular filtration rate; FBG, fasting blood glucose; HDLc, high-density lipoprotein cholesterol; hsCRP, high-sensitivity C-reactive protein; KIM-1, kidney injury molecule-1; LCN2, lipocalin-2; LDLc, low-density lipoprotein cholesterol; NGAL, neutrophil gelatinase-associated lipocalin; sAldo, serum aldosterone; SBP, systolic blood pressure; sCr, serum creatinine; sCysC, serum cystatin C; SDS-PAGE, sodium dodecyl sulphate-polyacrylamide gel electrophoresis; SIP24, super inducible protein 24; RT, retention time; TC, total cholesterol; TG, triglycerides; uAlb, urinary albumin; uAldo, urinary aldosterone; uCr, urinary creatinine; uHap, urinary haptoglobin; uKIM-1, urinary KIM-1; UPLC, Ultra Performance Liquid Chromatography.
